# Clinical Remarks on Wounds into Joints

**Published:** 1871-02

**Authors:** 


### Clinical Remarks on Wounds into Joints.

I wish to address you a few words on the subject of wounds into joints, at all times a serious accident, and, as regards the joint itself, not infrequently a fatal one. In case of doubt as to the joint being involved, it is hardly necessary to say, avoid all exploration. Nature will not permit any intrusion on or violence done to a joint. Peril almost certainly follows, If the joint be opened, and more especially if the wound through thc synovial membrane be large or contused, inflammation follows, and the outer wound, which may have shown a disposition to heal, opens. The margins inflame, or at least assume a red color; and a watery ichor first exudes from the joint, followed by pus. From the wound large and glassy granula-arise, which are eminently characteristic of a wounds into a joint. In this condition writers recommend a free incision into the cavity, under the idea that the joint is irretrievably lost. If the discharge of pus diminishes concurrently with increased pain and swelling of the joint, an incision, with a view to dilate the opening may by adviseable, but otherwise I do not think it is, because I am satisfied, from the observation of several cases, that the joint is occasionally perfectly recoverable. I can quote at least three cases in which pus was poured out from the knee-joint—in one of three days duration, in a second of ten days, and in a third of three weeks. In each of these cases the joints were perfectly restored to their natural functions. If this be so, will you not be careful in adopting what I can not but consider objectionable practice, that of a premature and fatal incision into a joint, which is yet susceptible of of cure by natural processes?—*Lancet, August 20th*, 1870.

